# The Use of Protein-DNA, Chromatin Immunoprecipitation, and Transcriptome Arrays to Describe Transcriptional Circuits in the Dehydrated Male Rat Hypothalamus

**DOI:** 10.1210/en.2014-1448

**Published:** 2014-08-21

**Authors:** Jing Qiu, Anna Kleineidam, Sabine Gouraud, Song Tieng Yao, Mingkwan Greenwood, See Ziau Hoe, Charles Hindmarch, David Murphy

**Affiliations:** School of Clinical Sciences (J.Q., A.K., S.G., S.T.Y., M.G., C.H., D.M.), University of Bristol, Bristol BS1 3NY, United Kingdom; and Department of Physiology (S.Z.H., C.H., D.M.), Faculty of Medicine, University of Malaya, Kuala Lumpur 50603, Malaysia

## Abstract

The supraoptic nucleus (SON) of the hypothalamus is responsible for maintaining osmotic stability in mammals through its elaboration of the antidiuretic hormone arginine vasopressin. Upon dehydration, the SON undergoes a function-related plasticity, which includes remodeling of morphology, electrical properties, and biosynthetic activity. This process occurs alongside alterations in steady state transcript levels, which might be mediated by changes in the activity of transcription factors. In order to identify which transcription factors might be involved in changing patterns of gene expression, an Affymetrix protein-DNA array analysis was carried out. Nuclear extracts of SON from dehydrated and control male rats were analyzed for binding to the 345 consensus DNA transcription factor binding sequences of the array. Statistical analysis revealed significant changes in binding to 26 consensus elements, of which EMSA confirmed increased binding to signal transducer and activator of transcription (Stat) 1/Stat3, cellular Myelocytomatosis virus-like cellular proto-oncogene (c-Myc)-Myc-associated factor X (Max), and pre-B cell leukemia transcription factor 1 sequences after dehydration. Focusing on c-Myc and Max, we used quantitative PCR to confirm previous transcriptomic analysis that had suggested an increase in *c-Myc*, but not *Max*, mRNA levels in the SON after dehydration, and we demonstrated c-Myc- and Max-like immunoreactivities in SON arginine vasopressin-expressing cells. Finally, by comparing new data obtained from Roche-NimbleGen chromatin immunoprecipitation arrays with previously published transcriptomic data, we have identified putative c-Myc target genes whose expression changes in the SON after dehydration. These include known c-Myc targets, such as the *Slc7a5* gene, which encodes the L-type amino acid transporter 1, ribosomal protein L24, histone deactylase 2, and the Rat sarcoma proto-oncogene (Ras)-related nuclear GTPase.

Osmotic stability is staunchly defended in mammals (1). Neuroendocrine responses to dehydration are mediated by the hypothalamo-neurohypophyseal system (HNS), a specialized part of the brain that is responsible for the highly regulated synthesis and secretion of the antidiuretic hormone arginine vasopressin (AVP). AVP is synthesized as part of a prepropeptide precursor in the cell bodies of the supraoptic nucleus (SON) and paraventricular nucleus (PVN) magnocellular neurones (MCNs) (2, 3). This precursor is processed during anterograde axonal transportation to nerve terminals located in the posterior pituitary gland, where biologically active AVP is stored until mobilized for secretion. The rise in plasma osmolality evoked by dehydration is detected by intrinsic MCN osmoreceptor mechanisms (4, 5), and by specialized osmoreceptive neurons in the circumventricular organs that project to the MCNs (6–10) and provide direct excitatory inputs (11) to shape the firing activity of MCNs (12, 13) for hormone secretion (14, 15). Upon release, AVP travels through the blood stream to specific receptor targets located in the kidney, where it increases the permeability of the collecting ducts to water, reducing the renal excretion of water, thus promoting water conservation (1).

The HNS also produces other neuropeptides in addition to AVP, for example, the closely related hormone oxytocin (OT), which, in addition to its well-known roles in parturition and lactation, is thought to have natriuretic activity at the level of the kidney (16). Single-cell RT-PCR enables AVP and OT transcripts to be detected in the same MCN (17), but the expression levels of each neuropeptide RNA differ by orders of magnitude. Only a small percentage (about 2%–3%) of MCNs express high, equivalent levels of both peptides (18), although the proportion increases after dehydration (19).

Dehydration evokes a dramatic remodeling of the SON and PVN (20, 21). A plethora of changes in the morphology, electrical properties, and biosynthetic and secretory activity of the HNS have all been described (22). Recently, microarray analysis has comprehensively described the changes in the rat SON transcriptome that accompany osmotic stimuli (23–25), and which may, at least in part, be responsible for shaping the resulting activity-dependent function-related plasticity.

We hypothesize that some of the changes seen in the HNS after dehydration are mediated by changes in the activity of transcription factors. We have thus used the Affymetrix combo protein-DNA array system to identify changes in the binding activity of transcription factors in response to water deprivation within the SON. Of the 26 protein-DNA associations thus identified, we have used EMSAs to validate 3 of these, namely cellular Myelocytomatosis virus-like cellular proto-oncogene (c-Myc)-Myc-associated factor X (Max), pre-B cell leukemia transcription factor 1 (Pbx1), and signal transducer and activator of transcription (Stat) 1/Stat3. For c-Myc-Max, we have closed the loop by comparing new data obtained from Roche-NimbleGen chromatin immunoprecipitation (ChIP) arrays (ChIP-Chip) with previously published transcriptomic data (23, 25) to identify putative c-Myc target genes whose expression changes in the SON after dehydration.

## Materials and Methods

### Animals

Adult male Sprague Dawley rats, weighing 225–250 g, were housed at a constant temperature of 22°C and a relative humidity of 50%–60% (vol/vol) under a 14-hour light, 10-hour dark cycle. All experiments were carried out under the licensing arrangements of the Animals (Scientific Procedures) Act (1986) with local ethics committee approval. Rats were given free access to food and tap water for at least 1 week before any experimentation. Dehydration involved complete fluid deprivation, with continued ad libitum access to food, for 3 days. Rats subjected to water deprivation decreased their food intake from approximately 30–35 g/d to less than 5 g/d by day 3 and body weight decreased by 15%.

### Nuclear protein preparation

For the protein-DNA array and EMSA, separate groups of 8 control animals and 8 dehydrated animals (2 animals pooled into 1 sample) were used. Animals were sacrificed by direct decapitation with a guillotine. The brain was rapidly removed from the cranium and placed in an ice-cold brain matrix (ASI Instruments). Two coronal brain slices of approximately 1-mm thickness were cut using the optic chiasm as reference. The coronal brain sections were placed on a Petri dish resting on a bed of ice, and the SON was visualized with the aid of a dissection microscope (Leica Microsystems) under ×10 magnification. The SON was identified as a highly vascularized semitranslucent tissue located immediately lateral of the optic chiasm in both right and left hemispheres of the brain and carefully dissected with a micro dissecting knife (catalog number 10055-12; Fine Science Tools) and fine Dumont forceps (Fine Science Tools). Tissues were stored at −80°C until homogenized with a minihomogenizer in 600 μL of lysis buffer (10mM NaCl, 10mM Tris HCl [pH 7.4], 1.5mM MgCl_2_, 0.5% vol/vol nonidet P-40, and protease inhibitor cocktail) (Sigma-Aldrich). Samples were incubated in ice for 30 minutes, then centrifuged for 2 minutes at 1000*g* at 4°C. The cytosolic supernatant was removed, then the pellet was resuspended in 200-μL wash buffer (10mM NaCl, 10mM Tris HCl [pH 7.4], 1.5mM MgCl_2_, 1% vol/vol nonidet P-40, 0.5% wt/vol sodium deoxycholate, and protease inhibitor cocktail) (Sigma-Aldrich), vortexed, incubated in ice for 5 minutes, and then centrifuged for 2 minutes at 1000*g* at 4°C. The wash step was repeated to remove residual cytosolic proteins. The pellet was resuspended in 50–100 μL of extraction buffer (20mM HEPES [pH 7.9], 0.4M NaCl, 1mM EDTA, 10% vol/vol glycerol, and 0.1mM dithiothreitol) containing 1× protease inhibitor cocktail (Sigma-Aldrich), vortexed, and incubated in ice for 2 hours on a plate shaker. The sample was finally centrifuged for 5 minutes at 15 000*g* at 4°C, and the nuclear protein supernatants were stored at −80°C.

### Protein-DNA array

The Affymetrix protein DNA combo array kit was designed to simultaneously analyze the binding activities of 345 transcription factors to their corresponding consensus DNA sequences. The array membrane is spotted with different consensus DNA sequences. Double-stranded DNA probes, 1 strand of which is biotinylated, are incubated with a nuclear extract sample to form protein-DNA complexes. The complexes are then separated from free DNA probes. Biotinylated DNA probes are then extracted from the protein-DNA complexes and are hybridized to the array membrane, the signals from which are proportional to the abundance of the corresponding DNA-binding protein in the original nuclear extract. Proprietary buffers were used except where stated. Probe mix (5 μL) was combined with 15–20 μg of SON nuclear extract (3–5 μg/μL), diluted in water to make a final volume of 20 μL, and then incubated for 30 minutes at 15°C to allow the DNA probes to bind to transcription factors.

Protein-bound probes were separated from unbound probes using a spin column kit. The spin column was washed with 500 μL of chilled 1× column incubation buffer and then centrifuged at 7000 rpm for 30 seconds at 4°C. A total of 20 μL of column incubation buffer were added to the transcription factor-probe mix, and the mixture was transferred into the center of the spin column and incubated in ice for 30 minutes. Unbound material was removed by centrifugation at 7000 rpm for 30 seconds at 4°C, and the flow through was discarded. The spin column was washed once with 1× column incubation buffer and 4 times with 1× column wash buffer. Finally, 60 μL of 1× column elution buffer were added to the center of the spin column and incubated at room temperature (RT) for 5 minutes. The spin column was then placed in a 1.5-mL microcentrifuge tube and centrifuged for 1 minute at 10 000 rpm at RT. The microcentrifuge tube, containing the bound probes, was kept on ice until proceeding to hybridization. The combo array membrane was wetted with water, then incubated in 3–5 mL of prewarmed hybridization buffer at 42°C for 2 hours. Just before hybridization, the eluted probes were denatured by heating at 95°C for 3 minutes, then quickly chilled in ice for 2 minutes. The denatured probes were added into the hybridization bottle and hybridized overnight in a rotating hybridization oven. The hybridization mixture was decanted, and the array membrane was washed with 50 mL of prewarmed hybridization wash I and then twice with hybridization wash II, each for 20 minutes at 42°C in a rotating hybridization oven. The membrane was incubated in 1× blocking buffer for 15 minutes with gentle shaking at RT. A total of 20 μL of streptavidin-horseradish peroxidase (HRP) conjugate were added to the blocking buffer and incubated for a further 15 minutes. The membrane was then washed 3 times at RT for 8 minutes with 1× wash buffer. The detection solution was prepared by mixing 200 μL of solution I, 200 μL of solution II, and 1.6 mL of solution III. The array membrane was placed on a plastic sheet, and the detection solution was pipetted onto the surface of the array membrane, which was then covered with another plastic sheet. After 5 minutes of incubation at RT, the excess working solution was removed, and the array membrane was exposed to HyperFilm enhanced chemiluminescence (ECL) (GE Healthcare Life Sciences). Images were scanned into a computer. The intensity of each spot, representing the binding activity of transcription factor(s) to consensus DNA sequences, was measured with ImageJ software (V1.48; National Institutes of Health) and presented as an arbitrary value. For absent spots, a minimum value of 10 was assigned in order to permit statistical analysis. Each dataset was normalized relative to the mean hybridization signal, based on the rationale that the total binding should be roughly the same in all samples in both control and dehydrated groups. Data were assessed using significance analysis of microarrays (SAMs) (26).

### Electrophoretic mobility shift assay

EMSAs were carried out using kits from Affymetrix in accordance with the manufacturers instructions. Double-stranded biotinylated DNA oligonucleotide EMSA probes were identical in sequence to the corresponding probe used in the protein-DNA array. Unlabeled probes of the same sequence were used in competition assays. The sequences of the EMSA probes used are: c-Myc-Max, 5′-GGA-AGC-AGA-CCA-CGT-GGT-CTG-CTT-CC (27); Stat1/Stat3, 5′-CTG-ATT-TCC-CCG-AAA-TGA-CGG (28); and Pbx1, 5′-CGA-ATT-GAT-GCA-CTA-ATT-GGA-G (29). In a final volume of 10 μL, nuclear extract (1 μg/μL) was combined with 1-μL 10× binding buffer, 1-μL poly(dI-dC) (1 μg/μL), and 1-μL (10 ng/μL) biotin-labeled probe, and the mixture was incubated at RT for 30 minutes. In competition experiments, 1-μL (300 ng/μL) of unlabeled probe was added 10 minutes before the addition of the labeled oligonucleotide. In supershift assays, 2-μL (2 μg/μL) rabbit polyclonal antibodies specific to Stat1 p84/p91 (E-23, sc-346 X; Santa Cruz Biotechnology, Inc) or 2-μL (2 μg/μL) Stat3 (H-190, sc-7179 X; Santa Cruz Biotechnology, Inc) were incubated in the binding reaction before the addition of biotin-labeled probe. Protein-DNA complexes were fractionated in a 15% (wt/vol) polyacrylamide gel in 0.5× TBE (0.05M Tris-borate [pH 8.9] and 1mM EDTA) for 1 hour at 120–140 V in an ice bath using a Mini Protean III electrophoresis apparatus (Bio-Rad). Complexes were then electrophoretically transferred to a nylon membrane (Roche) using a semidry electroblotting device for 30 minutes at 300 mA (Bio-Rad) and then fixed by UV cross-linking (0.120 J/cm^2^ for 3 min). The membrane was incubated with 10-mL 1× Affymetrix blocking solution for 15 minutes at RT on a rolling shaker. A total of 10 μL of streptavidin-HRP conjugate were added and incubation proceeded for another 15 minutes. The blocking solution was discarded, and the membrane was washed 4 times for 5 minutes with 10 mL of 1× Affymetrix washing buffer. The membrane was then incubated with 10 mL of 1× Affymetrix detection solution for 5 minutes at RT and laid on the surface of a plastic sheet. A total of 1 mL of Affymetrix working solution containing 200 μL of solution I, 200 μL of solution II, and 800 μL of solution III was pipetted onto the membrane, which was then covered with another plastic sheet and incubated for 5 minutes at RT. Excess substrate was removed, then the membrane was exposed to HyperFilm ECL (GE Healthcare Life Sciences). Images were scanned into a computer, and the intensity of each band was measured with ImageJ software and presented as an arbitrary value. Statistical significance was evaluated using the paired Student's *t* test. *P* < .05 was considered significant.

### TransAM c-Myc activity assay

SONs from control and dehydrated rats (n = 3, 2 rats pooled per experimental sample) were collected on ice, and nuclear extracts were immediately obtained using a Nuclear Extract kit (Active Motif), in accordance with the manufacturer's instructions. Protein concentration was determined using the ProStain protein quantification kit (Active Motive). The TransAM c-Myc kit (Active Motive), an ELISA-based assay, was use to quantitatively determine c-Myc DNA binding activity in 5 μg of these extracts, and in 5 μg of a standardized control extract from Jurket cells. This assay consists of a 96-well plate containing an immobilized oligonucleotide that contains the c-Myc consensus binding site (5′-CACGTG-3′). The active form of c-Myc contained in nuclear extract binds specifically to this oligonucleotide. After incubation with extract, c-Myc bound to DNA is detected by a TransAM c-Myc kit primary antibody that recognizes an epitope that is accessible after DNA binding. Addition of a secondary HRP-conjugated antibody provides a sensitive colorimetric readout that is quantified by spectrophotometry. Data were normalized with respect to the Jurket samples, then presented ± SD. Statistical significance was evaluated using the unpaired Student's *t* test. *P* < .05 was considered significant.

### RNA extraction and cDNA synthesis

Rats (n = 6 control, n = 6 dehydrated, 1 animal per sample) were stunned and decapitated, and brains were removed and immediately frozen in powdered dry ice. Brains were sectioned to 60-μm coronal section in a cryostat. Sections were mounted on glass slides and stained with 0.1% (wt/vol) toludine blue and then visualized on a light microscope until SON was visible. Using the optic chiasm for reference, samples were punched using a 1-mm micro punch (Fine Science Tools) from frozen brain slices and dispensed into 1.5-mL tubes kept on dry ice within the cryostat. After punching the slices were mounted on glass slides to confirm extraction of SON. Total RNA was extracted by combining Qiazol reagent with the RNeasy kit protocols (QIAGEN). A total of 1 mL of Qiazol reagent was rapidly added to frozen punch samples. After Qiazol phase separation with chloroform, 350 μL of the upper aqueous phase were removed, mixed with 1 volume of 70% (vol/vol) ethanol, and applied to RNeasy columns. The remaining steps were performed as recommended by the manufacturer. For cDNA synthesis, 200 ng of total RNA were reverse transcribed using the Quantitect reverse transcription kit (QIAGEN).

### Real-time quantitative PCR (qPCR) analysis

Steady state RNA levels in the SON were assessed by qPCR. Primers for *c-Myc* were predesigned by Quantitect Primer assays (QT00187201; QIAGEN). Primer for *Max* (5′-TGGAGAGCGACGAAGAGCAA-3′ and 5′-GCATTATGGTGAGCCCGTTTG-3′) and ribosomal protein L (RPL)19 (5′-GCGTCTGCAGCCATGAGTA-3′ and 5′-TGGCATTGGCGATTTCGTTG-3′) were synthesized by Eurofins Genomics. qPCR was performed using an ABI 7500 Sequence Detection System (ABI). The cDNA from the RT reaction was used as a template for subsequent qPCRs, which were carried out, in duplicate, in 25-μL reaction volumes using SYBR green master mix buffer (Roche). For relative quantification of gene expression, the 2^−ΔΔCT^ method was employed (30). Statistical analysis was performed using one-way ANOVA with Tukey's post hoc test. *P* < .05 was considered significant. *Rpl19* was used as a normalization control that is unchanged by dehydration. The average cycle threshold (CT) value of Rpl19 for the control group was 18.23 (SEM, ±0.07), whereas the average CT value of dehydrated group was 18.17 (SEM, ±0.04). Statistical analysis confirmed no significant difference (*P* = .45) in *Rpl19* after 3 days dehydration.

### Immunohistochemistry and immunofluorescence

Rats were anesthetized with sodium pentobarbitone (100 mg/kg ip) and transcardially perfused with 0.1M PBS (pH 7.4) followed by 4% (wt/vol) paraformaldehyde in 0.1M PBS. Brains were removed and postfixed overnight in 4% (wt/vol) paraformaldehyde followed by 30% (wt/vol) sucrose prepared in PBS. Coronal sections (40 μm) of the forebrain were cut on a cryostat, washed in 0.1M PBS (pH 7.4), then blocked in 5% (vol/vol) horse serum prepared in 0.1M PBS with 0.25% (vol/vol) Triton X-100 for 30 minutes, and then incubated with appropriate primary antibodies at 4°C for 48 hours. The sections were washed 3 times in PBS for 5 minutes and incubated with 1:500 dilution of appropriate biotinylated secondary antibody in 0.1M PBS with 0.25% (vol/vol) Triton X-100 for 1 hour at room temperature. The sections were washed 3 times for 5 minutes with PBS and incubated for 1 hour with Alexa Fluor 488 streptavidin-conjugated and Alexa Fluor 594 donkey antimouse or rabbit IgG (Invitrogen Life Technologies). After 3 washes with PBS, sections were mounted onto glass slides with 0.5% (wt/vol) gelatin and cover slipped with VectorShield hardset mounting media containing 4′,6-diamidino-2-phenylindole (DAPI) (Vector Laboratories Ltd). The next antibodies were used for immunostaining: rabbit polyclonal antibodies recognizing Max (C-124, sc-765, 1:2000; Santa Cruz Biotechnology, Inc) and c-Myc (1:2000, N-262, sc-764; Santa Cruz Biotechnology, Inc) and mouse monoclonal antibodies recognizing AVP neurophysin (NP-I, PS41; 1:100) and OT neurophysin (NP-II, PS38; 1:100) (31). Images were viewed and collected using a Leica SP5-AOBS confocal laser scanning microscope attached to a Leica DM I6000 inverted epifluorescence microscope (Wolfson Bioimaging Facility, University of Bristol).

### ChIP array (ChIP-Chip)

For in vivo analyses of c-Myc binding, the LowCell ChIP kit protein G (kch-maglow-G16; Diagenode) was used according to the manufacture's protocol and using proprietary reagents and buffers. ChIP was performed using a rabbit anti-c-Myc antibody (c-Myc N-262, sc-764; Santa Cruz Biotechnology, Inc). DNA shearing was performed using Soniprep 150 sonicator (3 rounds of 10 s on/30 s off on ice). Immunoprecipitated DNA was amplified using the GenomePlex Single Cell Whole Genome Amplification kit (WGA4) (Sigma-Aldrich). The samples were then purified and cleared of excess primers with the QIAquick PCR Purification kit (QIAGEN). Resulting samples were checked for quantity by NanoDrop spectrophotometry (Thermo Fisher Scientific) and quality with an Agilent Bioanalyzer (Agilent Technologies). All DNAs had a concentration over 250 ng/μL and totaled at least 3.5 μg. The A260/A280 ratio was over 1.7, and the A260/A230 ratio was over 1.6. ChIP-Chip was carried out by NimbleGen (Roche NimbleGen) using the RN34_Refseq_Prom_ChIP. Tiling of 15 287 promoter regions was based on refFlat file downloaded from the University of California at Santa Cruz on May 22, 2009. Each transcription start site was tiled 4280 bp upstream and 1070 bp downstream (n = 713 735, median probe spacing 100 bp). The ChIP-Chip assay was independently replicated 3 times. Data were conservatively filtered on the basis of being represented on all 3 arrays and having a mean peak score value (defined as the log2 ratio of the fourth highest probe in the peak) less than 1.0.

## Results

### Changes in transcription factor binding in the SON after dehydration

**Figure 1.**
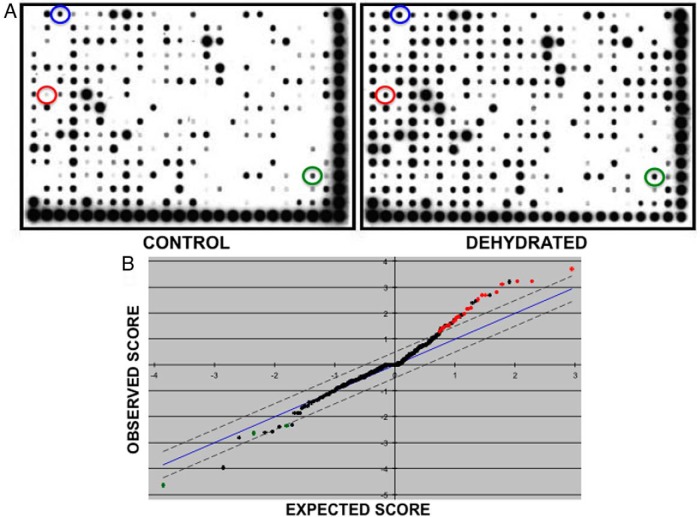
Affymetrix combo protein-DNA array analysis of control and dehydrated SON DNA-binding proteins. A, Representative image of a typical experiment. The array is spotted with 345 different consensus binding sequences. The degree of probe hybridization is directly proportional to the specific binding activities present in the nuclear extracts. Films were scanned, and the intensities of individual spots were measured using ImageJ software. The spots corresponding to Stat1/Stat3 (green), Pbx1 (blue), and c-Myc-Max (red) are circled. B, SAM plot. SAM analysis of 4 independent experimental replicates revealed 23 significant increases in protein-DNA binding activity in the dehydrated groups compared with the control groups (red dots), and 3 significantly reduced protein-DNA binding activities in the dehydrated groups compared with the control groups (green dots).

The Affymetrix combo protein-DNA array system was used to identify changes in the DNA binding activity of transcription factors in response to 3 days of water deprivation within the rat SON. Nuclear extracts of SON from control or dehydrated rats were analyzed for binding to the 345 consensus DNA transcription factor binding sequences of the array. The experiment was independently replicated 4 times (2 animals pooled per group). A representative experiment is shown in Figure 1A. Statistical analysis of the data was carried out using SAM (26). The SAM plot revealed 26 significant differences in protein-DNA binding between the control and dehydrated groups (Figure 1B). The significant changes are listed in Table 1.

**Table 1.** Changes in DNA Binding Activities in SON Nuclear Extracts from Dehydrated Compared with Control to Rats

Array Position	Binding Site Name	SAM Score	Fold Change
B5	CEF1	3.711419206	2.39910
J2	NF-E1 (YY1)	3.247889339	1.78143
G3	SIE	3.222224092	1.84766
G2	Myc-Max	3.123605079	2.47520
I3	Smad3/4	2.815971202	1.60153
I1	CREB	2.683277474	1.60469
G1	CDP	2.681437062	1.64696
C2	GRE	2.537374617	2.42972
A7	HFH-3	2.188579519	1.51920
B1	AP-2	2.170323778	2.07041
B6	Freac-2	2.145124972	2.20127
A1	AP1	1.971349655	3.95190
A5	CdxA/NKX2	1.854122612	1.81407
K14	SP1 ASP	1.83475338	2.60678
D8	NF-Y	1.825249587	1.65482
M22	Stat1/Stat3	1.729044915	2.12851
C22	PTF1	1.655612186	2.49453
I2	NFATc	1.523420984	2.44911
A3	Pbx1	1.505972905	1.54377
K6	GATA-4	1.450991788	1.53952
E11	EKLF (1)	1.437226509	1.60058
B21	N-rax BP	1.390028844	2.42782
H7	Ikaros	1.276618115	1.53800
O11	HOXD8 9 10	2.344140695	0.63798
M17	EGR1	2.628428552	0.58570
O13	PEBP2	4.650603144	0.60352

### Validation by EMSA and TransAM ELISA

**Figure 2.**
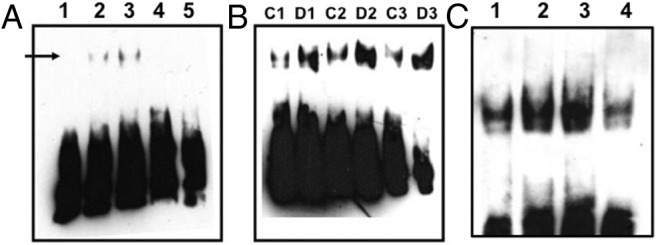
Stat1/Stat3 EMSA. Biotin-labeled double-stranded oligonucleotide probes were incubated with nuclear proteins extracted from control or dehydrated SON. After electrophoresis, complexes were transferred to a membrane for ECL detection. A, Lane 1, probe only; lane 2, probe with control SON nuclear extract; lane 3, probe with dehydrated nuclear extract; lane 4, probe, control SON nuclear extract, and an excess of unlabeled oligonucleotide probe; lane 5, probe, dehydrated SON nuclear extract, and an excess of unlabeled oligonucleotide probe. The arrow indicates the position of a specific complex. B, Three further independent EMSA experiments (control [C], 1–3; and dehydrated [D], 1–3) confirm an increase in Stat1/Stat3 complexes after dehydration. C, Supershift analysis with specific Stat1 or Stat3 antibodies reveals that the Stat1/Sta3 complex in the dehydrated SON contains Stat3 but not Stat1. Lane 1, EMSA with control extract; lane 2, EMSA with dehydrated extract; lane 3, supershift EMSA with dehydrated extract and Stat1 antibody; lane 4, supershift EMSA with dehydrated extract and Stat3 antibody. The specific complex is disrupted by the Stat3 antibody, not the Stat1 antibody.

**Figure 3.**
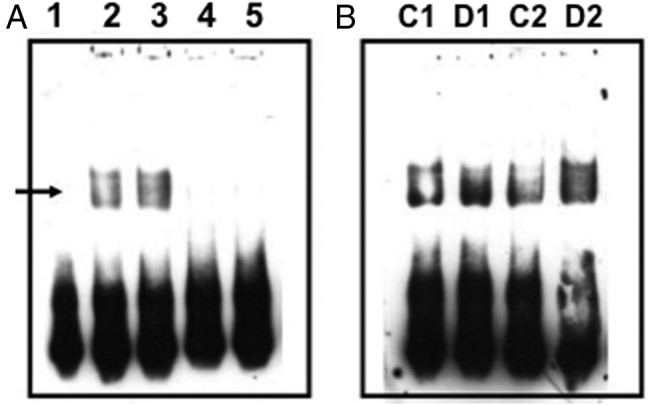
Pbx1 EMSA. Biotin-labeled double-stranded oligonucleotide probes were incubated with nuclear proteins extracted from control or dehydrated SON. After electrophoresis, complexes were transferred to a membrane for ECL detection. A, Lane 1, probe only; lane 2, probe with control SON nuclear extract; lane 3, probe with dehydrated nuclear extract; lane 4, probe, control SON nuclear extract, and an excess of unlabeled oligonucleotide probe; lane 5, probe, dehydrated SON nuclear extract, and an excess of unlabeled oligonucleotide probe. The arrow indicates the position of a specific complex. B, Further independent EMSA experiments (control [C], 1–2; and dehydrated [D], 1–2) confirm an increase in Pbx1 complexes after dehydration.

**Figure 4.**
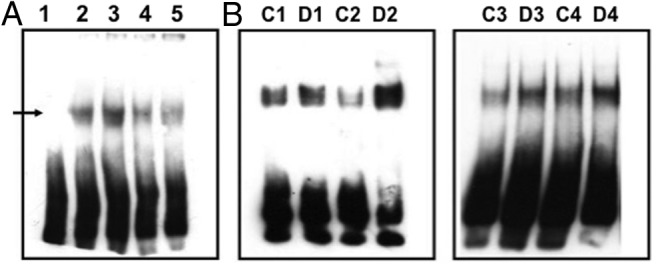
c-Myc-Max EMSA. Biotin-labeled double-stranded oligonucleotide probes were incubated with nuclear proteins extracted from control or dehydrated SON. After electrophoresis, complexes were transferred to a membrane for ECL detection. A, Lane 1, probe only; lane 2, probe with control SON nuclear extract; lane 3, probe with dehydrated nuclear extract; lane 4, probe, control SON nuclear extract, and an excess of unlabeled oligonucleotide probe; lane 5, probe, dehydrated SON nuclear extract, and an excess of unlabeled oligonucleotide probe. The arrow indicates the position of a specific complex. B, Four further independent EMSA experiments (control [C], 1–4; and dehydrated [D], 1–4) confirm an increase in c-Myc-Max complexes after dehydration.

EMSA was used to confirm increased binding to the Stat1/Stat3 (3.7-fold increase; *P* = .0027, n = 3) (Figure 2), c-Myc-Max (1.7-fold increase; *P* = .0413, n = 4) (Figure 3), and Pbx1 (1.3-fold increase; *P* = .0445, n = 3) (Figure 4) probes after dehydration. In all cases, probe binding was reduced by the addition of an excess of unlabeled probe (Figures 2A, 3A, and 4A). The presence of Stat3, but not Stat1, in the protein-Stat1/Stat3 probe complex was demonstrated by antibody supershift (Figure 2C). An increase in the activity of c-Myc in the dehydrated SON was confirmed by the use of the TransAM c-Myc activity ELISA assay (1.4-fold increase, ±0.31; *P* = .026, n = 7).

### Myc and Max expression in the SON

**Figure 5.**
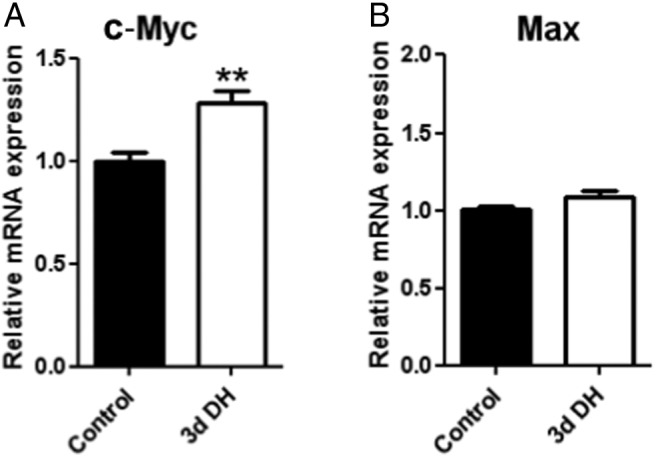
*c-Myc-* and *Max* mRNAs in the SON. Relative mRNA expression levels of (A) *Myc* and (B) *Max* in SON of 3 days of dehydrated compared with control rats (n = 6 each group). Error bar is ±SEM. **, *P* < .01.

**Figure 6.**
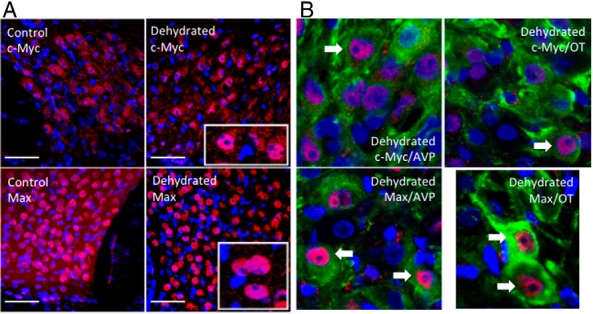
c-Myc- and Max- like proteins in the SON. A, c-Myc- and Max-like immunoreactivities are found in the SON of both control and dehydrated rats. c-Myc staining is both nuclear and cytosolic, whereas Max is mainly nuclear. DAPI nuclear labeling is revealed in blue. The scale bar is 50 μm. Insets show close up views of representative neurons. B, Immunocytochemistry reveals c-Myc-like and Max-like material in both AVP and OT cells of control and dehydrated SON. Antibody binding to c-Myc-like and Max-like antigens was detected with Alexa Fluor 594 (red). AVP or OT was detected with Alexa Fluor 488 (green). DAPI nuclear labeling is revealed in blue.

We have previously used Affymetrix GeneChip analysis to identify changes in the expression of mRNAs encoding transcription factors in the rat SON as a consequence of 3 days of dehydration (25). Examination of these datasets suggested a significant (∼1.64-fold) increase in *c-Myc* mRNAs but no change in the expression of *Max* transcripts. This has been confirmed by qPCR. *c-Myc* mRNA levels increased with dehydration (1.287 ± 0.058; *P* < .01, n = 6) (Figure 5), but *Max* transcript levels did not significantly change (1.085 ± 0.036; not significant, n = 6) (Figure 5). Both c-Myc- and Max-like immunoreactivities were found in the SON of both control and dehydrated rats (Figure 6A). c-Myc staining is both nuclear and cytosolic, whereas Max is mainly nuclear. Because the SON contains 2 distinct MCN populations, those that express AVP and those that express OT, we sought to identify which of these cell types express c-Myc or Max. c-Myc- and Max-like immunoreactivities are found in both AVP and OT expressing MCNs.

### Identification of putative c-Myc genomic targets in the SON

Roche NimbleGen promoter arrays were used to identify putative c-Myc target genes in the dehydrated rat SON. The ChIP-Chip assay was independently replicated 3 times. Data were conservatively filtered on the basis of being represented on all 3 arrays and having a mean peak score value <1.0 (Supplemental Table 1). In total, 675 genes were identified as having promoters that bind c-Myc. Comparison with transcriptome data (23, 25) revealed a subset of 116 genes with promoters that bind to c-Myc, and whose transcript is regulated in the SON after 72 hours of dehydration (Supplemental Table 1).

## Discussion

We have combined the use of 3 array platforms to describe transcriptional circuits in the SON of the rat. Firstly, we used Affymetrix protein-DNA arrays to identify changes in the specific binding of transcription factors to *cis*-elements contained within double-stranded oligonucleotides. Increased binding to 3 sequences (Stat1/Stat3, Pbx1, and c-Myc-Max) was confirmed by EMSA and, in the case of c-Myc-Max, TransAM ELISA. Because Affymetrix transcriptome array analysis had previously suggested that *c-Myc* was up-regulated at the level of mRNA level in the SON by dehydration, we focused on the c-Myc-Max heterodimer and confirmed that both c-Myc and Max are expressed in AVP- and OT-expressing MCNs in the SON and that *c-Myc*, but not *Max*, transcripts are up-regulated by dehydration. We used Roche NimbleGen ChIP-Chip promoter arrays to identify putative genomic c-Myc targets in the dehydrated male rat SON. Comparison of these data with previously published transcriptome catalogues obtained using Affymetrix GeneChip arrays (23, 25) revealed genes that are possibly up-regulated in the SON as a consequence of c-Myc action.

The Affymetrix protein-DNA array enables the identification of interactions between proteins in a nuclear extract with DNA sequence that has previously been defined as a binding site for a particular transcription factor or factors. Thus, the precise identity of the protein component of the complex is not known. Validation by EMSA and other techniques is thus required.

In total, SAM analysis of the Affymetrix protein-DNA array data revealed significant changes to the binding of 26 consensus binding sites; binding to 23 sequences is increased, whereas binding to 3 is decreased. Gratifyingly, some of these interactions have been previously observed to change in the osmotically stimulated SON.

Our protein-DNA array data suggested increased protein binding to cAMP response element-binding protein (CREB) sequences after dehydration. Consistent with these results, a previous study using immunohistochemistry with an antibody raised against phosphorylated CREB (pCREB) revealed that there was a significant increase in the number of pCREB-containing cells in the SON after hypertonic saline injection (32). However, using EMSA, although it was shown that constitutive pCREB binding was substantial in the SON, no consistently significant changes in binding activity after hypertonic saline treatment were reported (33). It has been suggested that CREB may be responsible for the osmotically stimulated increase in AVP gene transcription in the SON (34, 35). However, recent in vivo studies, injecting a recombinant adeno-associated virus expressing a dominant negative mutant form of CREB into rat SON, failed to significantly reduce AVP mRNA expression after hyperosmotic stimulation (36). We have recently reported that a related CREB/activating transcription factor transcription factor, CREB3L1, is dramatically up-regulated in the SON by an osmotic stimulus (37), and it may well be this protein that is binding to the CREB oligonucleotide on the protein-DNA array.

Another interaction identified in the protein-DNA array was with activator protein 1 (AP1) sequences. It has also been previously demonstrated that binding activity to AP1 *cis*-elements is increased in the osmotically stimulated SON (33, 38), concomitant with increases in the expression of RNAs encoding members of AP1 family of Finkel-Biskis-Jinkins murine osteogenic sarcoma virus-like cellular proto-oncogene (Fos) (eg, *c-Fos*) (39) and V-Jun Sarcoma Virus 17-like cellular proto-oncogene (Jun) proteins (eg, *JunD*) (40).

From the 26 significant interactions identified in the protein-DNA array, we narrowed our attention to 3 novel binding activities, namely Stat1/Stat3, Pbx1, and c-Myc-Max, all of which were confirmed by EMSA.

Out of the 26 significant interactions identified in the protein-DNA array, some have previously been described. Although this adds credence to our approach, we sought to understand novel interactions by focusing on 3 representative examples, namely Stat1/Stat3, Pbx1, and c-Myc-Max, all of which were confirmed by EMSA.

The Stat proteins are a family of cytoplasmic transcription factors that can be activated by distinct cytokines or growth factors and are involved in a variety of intracellular signaling events (41). Upon activation by phosphorylation, they form homodimers or heterodimers and translocate to the nucleus to mediate gene expression by interacting with cognate *cis*-acting elements in the genome (42). The Stat1/Stat3 consensus DNA-binding sequence, also known as the interferon-response element (pIRE/interferon regulatory factor 1–1), was originally shown to be responsible for the activation of interferon regulatory factor 1 by IL-6 or interferon-γ (43). Both Stat1 and Stat3 can bind to this sequence, either as homodimers or heterodimers. Thus, in order to determine which transcription factors bind to Stat1/Stat3 DNA sequence, supershift analyses with antibodies raised against Stat1 and Stat3 were performed (Figure 3), revealing that it is the latter factor that is activated in the dehydrated SON. It has been shown that IL-6 is markedly up-regulated in the SON, at both mRNA and protein levels, after 3 days of water deprivation (44). IL-6 has been shown to be coexpressed with AVP in the SON (44, 45) and has been linked to AVP release (46, 47). Interestingly, IL-6 is a potent activator of Stat3 in a variety of cell types (48), and intracerebroventricular injection of IL-6 induced nuclear translocation of Stat3 in neurons of both the SON and PVN (49). Further, it has been shown that Stat3 can activate the expression of AP1 proteins, such as c-Fos (50). It is thus possible that the increased binding of Stat3 to the Stat1/Stat3 consensus sequence in the SON after dehydration might be mediated by IL-6, which, in turn, might regulate AVP gene expression by transcriptionally activating the expression of transcription factors, such as c-Fos.

Pbx1 belongs to the PBX and Ceh20 (PBC) subclass of the 3-amino acid loop extension proteins characterized by an atypical homeodomain (51) that forms stable complexes with other homeodomain transcription factors, such as Homeobox (Hox) (52), altering Hox DNA binding affinity and transcriptional activity (53). We have previously used Affymetrix microarrays to catalogue the expression of transcription factor genes in the SON of euhydrated and dehydrated rats (23, 25). Mining of these data has revealed that, of the rat PBC genes, only *Pbx1* and *Pbx4* are expressed in the SON, and the level of expression does not change with dehydration, suggesting that the increased binding seen in the dehydrated SON is mediated by translational or posttranslational mechanisms. Further, we searched our transcriptome datasets (25) for potential homeodomain-containing Pbx1/Pbx4 partners; 2 were identified, putative homeodomain transcription factor 1, which appears to be up-regulated by dehydration, and homeodomain only protein X, which is down-regulated.

c-Myc is a transcription factor containing a number of defined functional domains. At the N terminus is a 150-amino acid transcriptional activation domain (TAD) (for review, see Ref. 54). At the C terminus is a composite basic (b)-helix-loop-helix (HLH)-leucine zipper (Zip) domain composed of 3 different elements, the b, the HLH, and the Zip regions. The function of this domain is to specify homo- or heterodimerization through the HLH-Zip region and interaction with DNA through the basic region. c-Myc, on its own, does not homodimerize and hence cannot affect transcription. Rather, c-Myc needs a partner, such as Max, which heterodimerizes with c-Myc via its own b-HLH-Zip motif to mediate Myc function (54). Both Max-Max homodimers and c-Myc-Max heterodimers bind to a consensus E-box sequence 5′-CACGTG-3′. However, Max does not have a TAD, thus DNA-bound Max-Max homodimers cannot activate transcription. c-Myc-Max heterodimers have a higher affinity for the 5′-CACGTG-3′ motif than Max-Max homodimers, and only c-Myc-Max heterodimers can activate transcription, through the c-Myc TAD (54). Examination of our SON transcriptome datasets (25) revealed expression of both *c-Myc* and *Max* in the euhydrated SON, but only *c-Myc* expression was increased by dehydration, observations now confirmed by qPCR (Figure 5). Further, we have shown that c-Myc- and Max-like immunoreactivities are found in the SON of both control and dehydrated rats (Figure 6). c-Myc staining is both nuclear and cytosolic, whereas Max is mainly nuclear. c-Myc and Max are found in both AVP and OT expressing MCNs. Thus, we suggest that c-Myc-Max heterodimers control gene expression in the SON and that, after dehydration, elevated levels of c-Myc will increase the amount of Myc-Max heterodimers relative to Max-Max homodimers, thus altering transcriptional responses. Roche NimbleGen promoter arrays were then used to identify the putative c-Myc genomic targets in the dehydrated rat SON, with a total of 675 genes being thus identified as having promoters that bind c-Myc. We note that in vivo occupancy of genomic binding sites does not always equate to transcriptional output. Indeed, it has been estimated that only 25% of mammalian transcription factor binding sites identified by ChIP in cells are linked to transcriptional activity (55). Expression profiling of the transcription factor-responsive transcriptome therefore remains key to understanding the physiological relevance of transcription factor binding. Comparison of our new ChIP array data with our previous transcriptome data (25) enabled the identification a subset of 116 genes that are apparently regulated in expression as a consequence of dehydration in the SON (Supplemental Table 1), perhaps through the action of c-Myc-Max binding. Most these genes are up-regulated, but some are down-regulated, and indeed, it is known that c-Myc can mediate transcriptional repression (56).

Many of the genes identified have previously reported to be regulated by c-Myc. Thus, for example, the increased biosynthesis of neuropeptide precursors in response to osmotic stimulation would demand increased importation of amino acid precursors, mediated by the product of the *Slc7a5* gene, the L-type amino acid transporter 1, a known c-Myc target (57). Similarly, a decrease in the level of the putative c-Myc target *Rpl24* (55) might mediate altered translation. At the level of the genome, a c-Myc-mediated repression of histone deactylase 2 expression (58) might provoke chromatin remodeling and subsequent changes in gene expression, with altered transport from nucleus to cytosol being mediated by the Rat sarcoma proto-oncogene (Ras)-related nuclear GTPase, a known c-Myc-regulated gene (59). Thus, although neither AVP nor OT appear to be direct c-Myc-Max targets, the function related plasticity in the dehydrated SON involves many changes that support increased hormone synthesis and secretion.

In summary, we have identified a subset of the proteome, corresponding to DNA-binding proteins that is altered in activity by dehydration, and have gone on to identify putative genomic targets of one of these transcription factors, namely c-Myc. Comparison with concurrent transcriptome changes identifies putative up-regulated c-Myc target genes. Thus, for the first time, we have “closed the loop” on a transcriptional circuit in the physiologically activated SON.
